# Insights into Gene Regulatory Networks in Chondrocytes

**DOI:** 10.3390/ijms20246324

**Published:** 2019-12-15

**Authors:** Hironori Hojo, Shinsuke Ohba

**Affiliations:** 1 Center for Disease Biology and Integrative Medicine, Graduate School of Medicine, The University of Tokyo, Tokyo 113-8655, Japan; hojo@tetrapod.t.u-tokyo.ac.jp; 2 Department of Bioengineering, Graduate School of Engineering, The University of Tokyo, Tokyo 113-8655, Japan; 3 Department of Cell Biology, Institute of Biomedical Sciences, Nagasaki University, Nagasaki 852-8588, Japan

**Keywords:** gene regulatory networks, next-generation sequencers, chondrogenesis, Sox9

## Abstract

Chondrogenesis is a key developmental process that molds the framework of our body and generates the skeletal tissues by coupling with osteogenesis. The developmental processes are well-coordinated by spatiotemporal gene expressions, which are hardwired with gene regulatory elements. Those elements exist as thousands of modules of DNA sequences on the genome. Transcription factors function as key regulatory proteins by binding to regulatory elements and recruiting cofactors. Over the past 30 years, extensive attempts have been made to identify gene regulatory mechanisms in chondrogenesis, mainly through biochemical approaches and genetics. More recently, newly developed next-generation sequencers (NGS) have identified thousands of gene regulatory elements on a genome scale, and provided novel insights into the multiple layers of gene regulatory mechanisms, including the modes of actions of transcription factors, post-translational histone modifications, chromatin accessibility, the concept of pioneer factors, and three-dimensional chromatin architecture. In this review, we summarize the studies that have improved our understanding of the gene regulatory mechanisms in chondrogenesis, from the historical studies to the more recent works using NGS. Finally, we consider the future perspectives, including efforts to improve our understanding of the gene regulatory landscape in chondrogenesis and potential applications to the treatment of chondrocyte-related diseases.

## 1. The Concept of Genomic Control of Development

The gene regulatory network is key to understanding how the genome encodes links to developmental processes, including cell fate specification, cell differentiation, and cell type-specific biological functions [1]. In 1957, Waddington introduced the concept of the “epigenetic landscape” as a link between phenotypes and gene regulation [2]. In his model, a cell chooses its fate by following the contours of the developmental landscape, just as a ball rolls down through valleys. In 1969, Britten and Davidson introduced a related theory of the gene regulatory network, in which the DNA sequence-specific regulation of gene expression contributes to the genomic control of developmental processes [3]. In 1981, a DNA regulatory element was identified in a 72 bp tandem repeat of SV40 DNA [4]. This element was shown to function as a cis-regulatory element activating the transcription of a cloned β-globin gene; the activation was observed even after changing the orientation or location of the sequence among several positions [4]. This type of regulatory element is defined as an enhancer, a noncoding DNA sequence that can drive the target gene expression by interacting with the promoter of the target gene, regardless of the distance, location, or orientation of the sequence relative to the promoter [5]. Trans-proteins, such as transcription factors (TFs), bind to the enhancer elements to drive the gene expression through the enhancer–promoter action.

## 2. The Discovery of Cis-Trans Regulatory Mechanisms in Chondrocytes

Chondrogenesis is a key developmental process in endochondral ossification, a mode of ossification observed in most skeletal tissues in mammals. During endochondral ossification, mesenchymal cells are condensed and differentiated into mitotic chondrocytes that deposit extracellular matrix to make cartilage molds. During this process, chondrocytes line up to form columnar structures. Subsequently, the cells transit to a post-mitotic state and differentiate into prehypertrophic and hypertrophic chondrocytes. Hypertrophic chondrocytes secrete osteogenic factors, which are triggers of osteogenesis. Prior to the osteogenic input, bipotential progenitors exist in the perichondrium, a thin layer of cells originating from the condensed mesenchyme and located at the periphery of cartilage. Once the progenitors receive the input, they are specified into osteoblast precursors. They mature into osteoblasts depositing bone matrix, which form the bone collar. Osteoblast precursors also invade the mineralized cartilage with vascular tissues to replace the mineralized cartilage with bone matrix.

In an effort to better understand the gene regulatory network in chondrogenesis, numerous studies have been performed on cis-trans regulation, with a particular focus on identifying 1) cis-regulatory elements related to chondrogenesis, and 2) trans-proteins that act on the regulatory elements and play pivotal roles in chondrogenesis. To this end, regulatory elements of *collagen type II alpha 1 chain* (*Col2a1*) have been studied, given the abundancy and specificity of *Col2a1* expression in chondrocytes. The first report on this subject was made in 1986 and identified regulatory elements in the mouse *Col2a1* promoter [6]. Next, in 1987, an enhancer element was identified in a rat *Col2a1* genomic region [7]. In both these studies, in vitro reporter assays were conducted using the chloramphenicol acetyltransferase (CAT) gene fused with several *Col2a1* genomic regions and the basal promoter. In the 1987 study, a series of reporter assays with different sizes of genomic fragments in different cell-types revealed that a 620 bp fragment in *Col2a1* intron 1 had an enhancer property [7]. The reporter activation was observed regardless of the orientation of the sequence or its position between the element and CAT gene. The reporter activity was enhanced upon chondrocyte differentiation in limb bud cells, whereas it was very low in nonskeletal cells [7]. These results indicated that the 620 bp fragment was a chondrocyte-specific enhancer.

Subsequent studies further provided mechanistic insights into *Col2a1* transcription. First, an 18 bp DNA element in the chondrocyte-specific mouse *Col2a1* intron 1 enhancer was shown to be sufficient for chondrocyte-specific enhancer activity [8]. Tandem copies of the element promoted the reporter activity in chondrocytes but not in fibroblasts; transgenic reporter mice harboring this tandem repeat demonstrated chondrocyte-specific reporter activities [8]. Second, gel shift assays (electrophoretic mobility shift assay: EMSA) revealed that nuclear proteins extracted from chondrocytes, but not those taken from other cell types, were bound to the identified enhancer element, indicating that a chondrocyte-specific trans-protein was involved in the enhancer regulation [8]. Importantly, Sox9 was later identified as the protein that bound to the enhancer element [9,10,11].

Sox9 is characterized by a high-mobility-group (HMG) domain that is closely related to the Y chromosome-encoded testis-determining factor SRY. Overexpression of Sox9 enhanced the activity of the reporter driven by the *Col2a1* enhancer element; the activity was abolished by mutations in consensus HMG domain sites in the enhancer [9]. The requirement of Sox9 binding was also confirmed in vivo: Reporter transgenic mice carrying the mutation in the Sox9-binding sites in the *Col2a1* enhancer lost the chondrocyte-specific activity, whereas those with the wild-type sequence kept the activity [10]. Ectopic expression of Sox9 enhanced the expression of both a *COL2A1*-driven reporter gene and the endogenous *Col2a1* gene in transgenic mice [10]. Indeed, expressions of *Sox9* and *Col2a1* were highly overlapped throughout the body during mouse development [11]. Collectively, these findings demonstrated that Sox9 functions as a trans-activator to induce *Col2a1* expression through binding to the *Col2a1* enhancer. In addition to *Col2a1*, other regulatory elements of chondrocyte-related genes, including *Col9α1*, *Col10α1*, *Col11α2*, *Col27α1*, *Hapln1*, *Matn1*, and *Acan*, were identified by similar approaches [12,13].

Sox9 was discovered by human genetic studies: Mutations in human SOX9 were associated with campomelic dysplasia (CD), which is characterized by skeletal malformation and XY sex reversal [14,15]. A deeper understanding of the function of Sox9 in chondrogenesis was obtained by mouse genetic studies. Analyses of *Sox9*^–/–^/WT chimeric mice revealed that *Sox9*^–/–^ cells did not contribute to mesenchymal condensation [16]. *Sox9* deletion in limb buds and in *Col2a1*-expressing early chondrocytes led to a lack of limbs and severe chondrodysplasia, respectively [17]. The broad removal of *Sox9* within both proliferating and prehypertrophic chondrocytes prevented hypertrophic progression in mice [18,19], whereas overexpression of Sox9 in chondrocytes caused a delay of hypertrophy [20]. Thus, Sox9 is necessary for (1) mesenchymal condensation, (2) the survival, proliferation, and proper differentiation of chondrocytes, and (3) maintenance of the growth plate. Other key trans-activators in chondrocytes were also discovered mainly by genetic studies. These include the family members of Fox, Runx, MEF2, Nkx, Arid, AP-1, Gli, and so on. Please refer to two recent reviews for details [21,22].

## 3. Genome-Scale Analysis of Sox9 Action in Chondrogenesis

**Figure 1:**
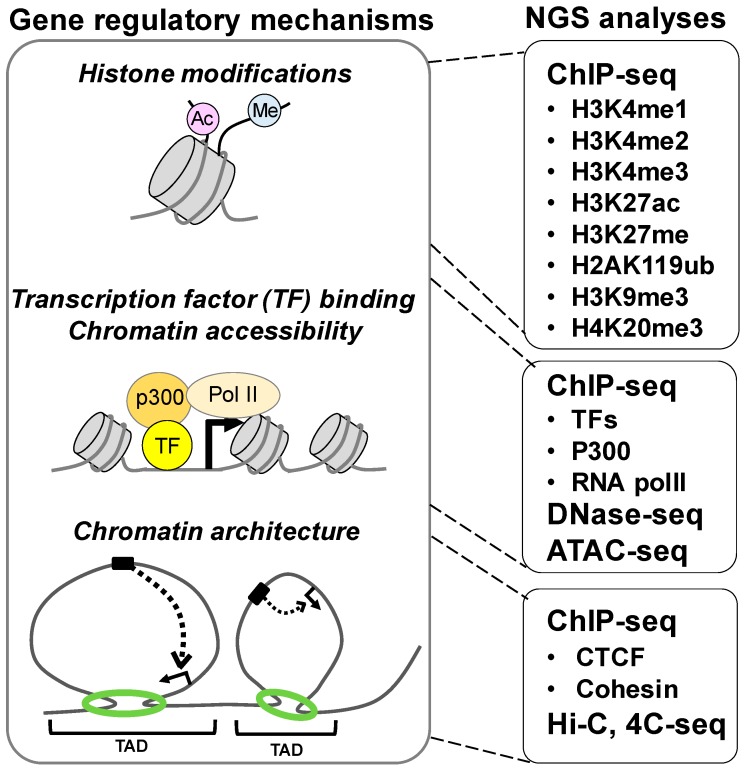
Multiple layers of the gene regulatory mechanism addressed by next-generation sequencers (NGS) analyses. Gene regulation is controlled at various scales of size, from histone modifications (left panel, top) and the binding of transcription factors (TFs) in chromatin-accessible regions (left panel, middle) to the topological organization of chromatin (right panel, bottom). Various NGS analyses have been performed to address each level of gene regulation (right panel). H3 and H4 refer to histone H3 and H4, respectively; K to the lysine residue involved; me1, me2, and me3 to mono-, di-, and tri-methylation, respectively; and ac and ub to acetylation and ubiquitination, respectively. Generally, H3K4me1 and H3K27ac mark active enhancers; H3K4me2 marks both active enhancers and transcription start sites (TSS); H3K4me3 marks TSS; H3K27me3 and H2AK119ub mark silenced enhancers and promoters, respectively; and H3K9me3 and H4K20me3 mark heterochromatin. Chromatin immunoprecipitation followed by sequencing (ChIP-seq) analyses for TFs have identified binding regions of the TFs on a genome scale. Upon TF binding, cofactors such as p300 are recruited, and transcription is initiated with RNA polymerase II. DNase I hypersensitive sites sequencing (DNase-seq) and the assay for transposase-accessible chromatin using sequencing (ATAC-seq) mark chromatin-accessible regions. Chromosome conformation capture analyses such as 4C-seq and high-throughput chromosome conformation capture (Hi-C) address the three-dimensional chromatin architecture and topologically associated domains (TADs). CTCF and cohesin are involved in the looping of chromatin structures.

Genome-scale studies with next-generation sequencers (NGS) have provided novel insights into the role of the gene regulatory network in development, shifting interest from local gene regulations to multiple layers of gene regulations governing aspects of development ranging from global dynamics of the regulatory landscape to three-dimensional chromatin architecture [23] (Figure 1). In the remainder of this paper, we review the recent progress in our understanding driven by NGS analyses and discuss insights into the gene regulatory network underlying chondrocyte development and chondrocyte-related diseases.

**Figure 2:**
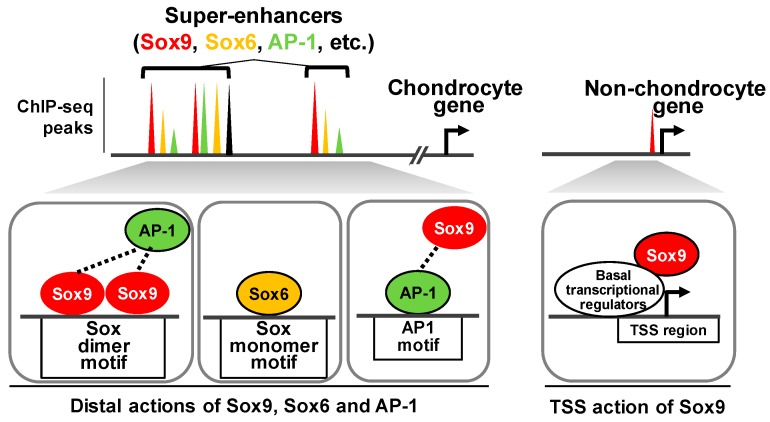
Key transcription factor-mediated gene regulatory mechanisms in chondrocytes identified by ChIP-seq studies. In ChIP-seq studies, the binding of a TF is detected as a “peak.” Several lines of evidence demonstrate that multiple TFs, including Sox9, Sox6, and activator protein-1 (AP-1), form “super-enhancer-like” clusters to regulate chondrocyte-distinct gene expression in the chondrocyte genome. These clusters are mainly located far from transcription start sites (TSS). In these clusters, Sox9 homodimers bind to Sox dimer motifs, whereas Sox6 binds to Sox monomer motifs. AP-1 binds to its consensus motifs, although AP-1 can cooperate with Sox9 through physical interactions. Sox9 also engages in the transcription of non-chondrocyte-related genes around TSSs; at these sites, Sox9 does not bind to the Sox motif, but instead indirectly associates with the genome through interactions with basal transcriptional regulators.

Local gene regulation, as exemplified by the Sox9-driven *Col2a1* regulation described above, is a good model to understand Sox9 action in chondrocytes. However, it is not clear how relevant this model is to the mode of Sox9 action on the chondrocyte genome and whether Sox9-bound enhancers are associated with chondrocyte-distinct functions. NGS studies of chromatin immunoprecipitation followed by sequencing (ChIP-seq) for Sox9 and a series of histone modifications revealed the genome-scale action of Sox9 in mouse primary rib chondrocytes [24] and the rat chondrosarcoma cell line, which retains characteristics of growth plate chondrocytes [25,26] (Figure 2). First, the actions of Sox9 in chondrocytes were divided into two modes, depending on the distance from transcription start sites (TSS) of the nearest gene: The TSS-associated actions and the distal actions [24]. The former was defined as Sox9 actions within a 500 bp window from the nearest TSS. In this mode, Sox9 is likely to associate with TSS regions of nonskeletal genes, including those associated with general cell activities, where Sox9 acts on the genome indirectly through protein–protein interactions with the basal transcriptional complex, such as p300 [27]. Given that this mode of action was positively correlated with the target gene expressions [24], Sox9 is likely involved in the transcriptional machinery to enhance the expression of these nonskeletal genes.

The other mode of Sox9 action comprises the distal actions taking place more than 500 bp away from the nearest TSS; this mode is highly associated with genes related to skeletal development and chondrocyte differentiation. The targets of this mode include cartilage matrix proteins, such as *Col2a1*, *Col9α1*, *Acan*, and *Matn1*, and transcriptional regulators in chondrogenesis, such as *Sox5* and *Sox6*, as well as *Sox9* itself. Sox9 has been shown to bind to tissue-specific active enhancers, which are marked by histone modifications including H3K4me2 and H3K27ac, and the transcriptional cofactor p300. Notably, Sox9 bindings show super-enhancer-like profiles [24,26]. Super-enhancers are defined as clusters of enhancers bound by master transcription factors with high densities of coactivators [28]. The highly condensed clusters are thought to be formed by the phase separation of coactivators [29,30]. Although there are no specific markers for super-enhancers at present, these enhancer clusters are likely to drive the robust expression of genes that play crucial roles in cell identities. These facts suggest that Sox9 is a main component of super-enhancers to define the chondrocyte identity.

*De novo* motif analysis in the Sox9 ChIP-seq studies has provided further genome-wide support for the mode of Sox9 action proposed by previous studies [31]: Sox9 acts as a homodimer through Sox dimer motifs, with each pair of Sox motifs oriented head-to-head with a three- or four-nucleotide spacer. It is of note that the recovered Sox9-binding motif showed extensive sequence degeneracy/variations. A set of the most frequently present nucleotides in each position within the degenerative Sox9 motifs was considered a Sox9 optimal motif. Importantly, Sox9 motifs present at Sox9-binding regions have a lower affinity to Sox9 than the optimal motif. Thus, Sox9 may favor suboptimal binding to genomic DNA in order to enable a flexible response, but utilize multiple bindings to enhancer clusters. This may be how Sox9 assures the high expression level of target genes and defines the chondrocyte lineage. The importance of suboptimal motifs for proper gene expression was shown in the neural plate-specific Otx-a enhancer in Ciona [32]. Motifs for GATA transcription factors and erythroblast transformation specific (ETS) domain transcription factors in the enhancer were not optimal on the genome. Optimization of the motif sequence or spacing caused stronger but ectopic enhancer activities, suggesting that suboptimized enhancers with weaker activities are required to restrict the activities to within specific tissues. Thus, suboptimal motifs may ensure the proper amounts and sites of enhancer activation, without changing the amount of input, i.e., the expression of regulatory proteins in the enhancers [33]. This finding also supports the idea that enhancers have evolved to achieve a spatiotemporal pattern of enhancer activities, rather than robustness of the activities.

## 4. Cooperative Actions of Multiple Transcription Factors to Establish a Chondrocyte Program

Gene-regulatory elements highly enrich multiple motifs of transcriptional regulators; the activation of such elements requires the binding of combinatorial TFs, including both lineage-specific master TFs and effectors of the signaling pathway [33,34]. Thus, understanding the cooperative actions of multiple TFs on the genome is key to understanding the enhancer landscape.

Sox5 and Sox6 are members of the Sox family that are co-expressed with Sox9 in chondrocytes [13]. These two factors share a highly conserved HMG domain with Sox9, although both lack a transactivation domain [13]. Loss-of-function analysis showed their pivotal but redundant roles in chondrogenesis [35]; a gain-of-function analysis of Sox5, Sox6, and Sox9 revealed that the trio, called the Sox trio, was necessary and sufficient to induce chondrocyte differentiation from either mouse embryonic stem cells or nonskeletal fibroblasts [36]. The cooperative action was also supported by a genome-wide analysis. A ChIP-seq study for Sox6 and Sox9 in a chondrocyte cell line revealed that their binding profiles were highly overlapped, particularly in chondrocyte super-enhancers. This indicates that these two Sox family members function together on super-enhancers in cartilage development [26]. On the other hand, the molecular mechanism underlying the interactions of Sox6 and Sox9 on DNA remains to be clarified. *De novo* motif analysis on ChIP-seq data and other biochemical assays confirmed that Sox6 favored Sox monomer motifs that are distinct from Sox9-binding motifs, i.e., Sox dimer motifs [26]. Given that no physical interactions were observed between Sox9 and Sox5/Sox6 [13], those factors might either bind to the genome independently or form a large transcriptional complex that has not been detected yet.

Potential interactions between Sox9 and non-Sox family members were suggested by the enrichment of their motifs in de novo motif analyses in Sox9 ChIP-seq studies [24]. One such interaction was recently identified between Sox9 and the transcription factors of activator protein-1 (AP-1) [37]. AP-1 is primarily composed of dimeric complexes of the Jun family, including Jun, Junb, and Jund, and the Fos family, including c-Fos, Fosb, Fosl1, and Fosl2. Chondrocyte-specific *Fosl2*-deficient mice showed a reduction in the hypertrophic chondrocyte zone and in mineralized hypertrophic cartilage matrix [38]; chondrocyte-specific *Jun*-deficient mice exhibited delayed chondrocyte hypertrophy specifically in the baso-occipital bone [39]. The ChIP-seq for Jun showed that the Sox9-binding regions were mostly shared with those of Jun on the chondrocyte genome [37]. The distribution of Sox9-binding motifs and AP-1-binding motifs in the shared regions suggests two distinct interacting modes: Direct binding of each factor to the same enhancers, and protein–protein interactions within AP-1- and Sox9-containing complexes. Biochemical analyses indicate that the direct co-binding of both factors to target motifs enhances target gene transcription, whereas protein –protein interactions of these attenuate the transactivations. Given the prehypertrophic chondrocyte-specific expression of Jun and Fosl2 and the positive actions of AP-1 and Sox9 on chondrocyte hypertrophy, the combined action of their two interacting modes on multiple enhancers may help to fine-tune the expression of target genes in the process.

The zinc finger Gli TFs are another cooperative factor of Sox9 in chondrocytes. Gli1, Gli2, and Gli3 are thought to mediate transcriptional responses to Hedgehog (Hh) input. In the growth plate, Indian hedgehog (Ihh), an Hh ligand, is expressed in prehypertrophic and hypertrophic chondrocytes. The Ihh-Gli axis plays crucial roles in chondrocyte differentiation and proliferation through a negative feedback loop formed with parathyroid hormone-related peptide (PTHrP) and by directly acting on chondrocytes, respectively [21]. Integrative analysis of the ChIP-seq data of Sox9 binding in mouse rib chondrocytes and the data on Gli1 and Gli3 binding in E11.5 mouse limb bud cells revealed that Sox9 and Gli cooperatively regulate common target genes in proliferating chondrocytes, including *Trps1*, *Sox9*, *Sox5*, *Sox6*, *Col2a1*, and *Ptch1* [40].

In addition to the factors described above, many proteins have been reported to regulate chondrogenesis; some of them form transcriptional modules. These include TFs acting as effectors of signaling pathways, co-factors, and chromatin modifiers (for review, see [21,22]). However, further studies will be needed to elucidate their precise mechanisms. Given that chondrocytes are dynamically changed during differentiation in terms of both morphologies and gene expressions, genome-scale analyses of the stage-specific interactions will provide the long-sought framework for the gene regulatory network in chondrogenesis.

## 5. Pioneer Factors for Establishing Cell Type-Distinct Gene Regulatory Networks

As described above, distinct combinations of TFs are crucial for cell fate determination. How do these combinatorial actions establish the cell type-specific regulatory state through the activations of numerous enhancers? Although there are millions of DNA sequences with the potential to act as enhancers on the genome, only a relatively small subset of genomic sequences can be functionally active in any given cell type; most of the genomic regions remain inaccessible due to nucleosome occupancy. How do specific genomic regions become accessible to be functionally active? A model using the concept of pioneer factors was proposed to answer this question, where pioneer factors were defined as a subset of master TFs that possess the unique ability to overcome the nucleosome occupancy [41]. In the model, pioneer factors bind to the inaccessible genomic regions and recruit other TFs, cofactors, and chromatin remodelers that make the underlying DNA more accessible to transcriptional machinery, and initiate cell type-specific gene expression programs. Liu et al. addressed whether Sox9 acts as a pioneer factor in chondrogenesis [42]. By profiling histone modifications and the transcriptome in limb buds of wild-type and *Sox9*-deficient mouse embryos, they revealed that Sox9 was involved in the epigenetic changes to some extent, but overall, it was dispensable for the initiation of chromatin dynamics and the corresponding gene expressions [42]. Given that Sox9 was reported to function as a pioneer factor in hair follicles [43], the requirements of Sox9 might be different between cell types, or other redundant factors may compensate for the Sox9 function in chondrogenesis. Identifying pioneer factors will thus be key to understanding how the chondrocyte fates are determined.

## 6. Higher-Order Gene Regulatory Machineries

The modeling of three-dimensional chromatin architecture has provided a new framework for understanding gene regulatory networks. Recently developed methods of chromosome conformation capture analyses such as 4C-seq and Hi-C technology have revealed that spatiotemporal gene expressions are achieved by complicated cis-trans actions from multiple enhancers; the sum of cell-type- or tissue-distinct activities of each regulatory element leads to the global expression pattern of a gene [44]. 4C-seq, reporter assays, and functional analysis with enhancers revealed that *Ihh* transcription was regulated by an ensemble of multipartite enhancers. Individual enhancer activities had different tissue specificities; the deletion of enhancers caused malformation of skeletal tissues, presumably due to a decreased expression of *Ihh*, in an additive manner of the deleted enhancers [45]. Consistent with this, an increase in the copy number of enhancers resulted in increased gene expression [45]. Functional redundancy of the enhancers was also shown in multiple gene regulations in mouse limb development [46]. In this study, the deletion of a single enhancer led to no significant change in limb morphology, whereas the removal of pairs of enhancers, which shared the same target gene, resulted in discernible phenotypes. The study also examined the combinatorial effect of enhancer deletion and the enhancer’s target gene deletion on limb development. Importantly, even the deletion of a single enhancer caused limb abnormalities, when one copy of its target gene was deleted. Deletions of two enhancers with the heterozygous target gene deletion resulted in a more severe phenotype [46]. These results suggest that functional redundancy is conferred by the additive effects of enhancers on gene expression levels.

Hi-C analysis demonstrates that many eukaryotic genomes are segmented into self-interacting domains called topologically associated domains (TADs) [47]. This compartmentalization restricts the interaction between cis-regulatory elements and the target basal promoter within the TAD [47]. Importantly, recent studies revealed that some genetic diseases are caused by the disruption of this machinery. Two recent studies demonstrated that a structural variant of the genome leading to the disruption of TAD boundaries underlies certain genetic diseases [48,49]. Lupianez et al. identified that the disruption of TAD boundaries flanking the EPHA4 gene is associated with congenital limb malformation in humans [48]. Mutant mice that recapitulate the human genome structural variant showed pathological phenotypes similar to those of human diseases, accompanied with the abnormality of enhancer–promoter interactions, resulting in ectopic expression of the adjacent (target) genes [48]. These studies highlight the association of the higher-order gene regulatory network with genetic diseases.

## 7. Gene Regulatory Networks Underlying the Association of Human Genetic Variants with Chondrocyte-Related Diseases

As the disruption of TADs is associated with diseases, mutations in noncoding regions also underlie human genetic diseases in skeletal tissues. Genome-wide association studies (GWAS) revealed that more than 90% of disease-associated loci identified so far were localized outside of protein-coding regions. One study revealed that mutations in the *Sonic hedgehog* enhancer, which is located 1 Mb away from the TSS, were related to polydactyly [50]. Another study showed that a variant in the *GDF5* regulatory element may be related to ancient selection in humans, which influences human growth and, potentially, osteoarthritis risk [51]. A more recent study integrated epigenetic profiles with GWAS to identify a causal mechanism in human height variants [52]. In that study, open chromatin regions in growth plate chondrocytes were highly associated with genes related to chondrocyte and skeletal biology. Importantly, the open chromatin regions were also enriched at height GWAS loci, where binding motifs of chondrocyte-related TFs were enriched. For example, regulatory regions of *CHSY1*, where a candidate causal variant (rs9920291) was located, were overlapped with open chromatin regions in chondrocytes, and a variant of the *HOXD13*-binding motif was involved in the enhancer activity of the regions [52]. Given that epigenetic profiles have been investigated in human diseases, including common diseases and osteoarthritis [53,54], integrative analysis of these profiles with the GWAS- and TF-binding profiles in a given cell type should provide mechanistic insight into the gene regulatory networks underlying the association of causal variants with diseases.

## 8. Concluding Remarks and Future Perspective

As we have shown in this review, emerging genome-wide studies have provided insights into gene regulatory networks in chondrocytes by organizing multilayers of regulatory mechanisms. However, one limitation would be that these studies only provided a “snapshot” profile of TF bindings or the chromatin status in cells, which are potentially heterogeneous populations. Given that the gene regulatory networks are dramatically changed during cell fate specification and cell differentiation, the next challenge will be to identify the dynamic landscape during chondrogenesis. Building on the findings of previous studies in mouse in vivo chondrocytes [24,37], we recently clarified the dynamic change in open chromatin signatures and bindings of transcriptional regulators in in vitro chondrocyte differentiation from human pluripotent stem cells [55]. Detailed analyses on the distinct types of in vivo cells, by taking advantage of cell sorting from reporter mice and recently developed single-cell technologies such as single-cell assays for transposase-accessible chromatin using sequencing (scATAC-seq) [56], will be useful to dissect the developmental stage-specific gene regulatory network. In addition, detailed transcriptional profiling in distinct cell types can be linked to the gene regulatory network during cartilage formation and pathogenesis. Single-cell RNA-seq (scRNA-seq) analyses in growth plate chondrocytes [57,58], skeletal development [59], and cartilage pathogenesis [60] may provide insights into the dynamic molecular link at the single-cell level.

In addition to identifying key genomic regions, the CRISPR/Cas9 technology enables manipulation of epigenetic states at given genomic loci in order to test their therapeutic potentials. For example, the guide-RNA-targeting of specific loci with nuclease-null, deactivated Cas9 (dCas9) fused to the catalytic core of the acetyltransferase p300 specifically activated the targeted enhancers [61], whereas that with dCas9 fused to either histone demethylase or Krüppel associated box (KRAB) domain, a potent repressor domain, suppressed their activity [62,63]. Given its specificity in the local gene regulatory elements and avoidance of exogenous gene integration into the genome, epigenome editing will be an attractive approach for the treatment of genetic disorders and/or tissue regeneration. In addition, a functional enhancer screening was recently conducted by combining epigenome editing with scRNA-seq [64]. In this study, 5920 human candidate enhancers were perturbed by a CRISPR interference system with dCas9-KRAB. The effect of the enhancer perturbation on putative target gene expressions was assessed by profiling 254,974 single-cell transcriptomes, resulting in the identification of 470 highly confident enhancer-gene pairs. Moreover, Mochizuki et al. conducted a combinatorial assay of CRISPR screening with mass spectrometry, focusing on *Sox9* gene regulatory elements [65]. They identified a chondrocyte-specific Sox9 enhancer that was activated by Stat3 [65]. These screening technologies are powerful tools in the field and will be extensively used with higher throughput.
